# Introducing of the First DCD Kidney Transplantation Program in Poland

**DOI:** 10.1155/2019/6302153

**Published:** 2019-02-28

**Authors:** Honorata Stadnik, Tomasz Małkiewicz, Michał Stronka, Lucyna Cichańska, Łukasz Kawalec, Mateusz Puślecki, Marcin Ligowski, Marcin Zieliński, Marek Karczewski

**Affiliations:** ^1^Department of General and Transplant Surgery, Clinical Hospital of Poznan University of Medical Sciences, 60-355 Przybyszewski St. 49, Poland; ^2^Anesthesiology and Intensive Care Unit, Clinical Hospital of Poznan University of Medical Sciences, 60-780 Grunwaldzka St. 16/18, Poland; ^3^Department of Cardiac Surgery and Transplantology, Clinical Hospital of Poznan University of Medical Sciences, 61-848 Długa St. 1/2, Poland; ^4^CEO Emergency Ambulance Service Poznan, 60-346 Rycerska St. 10, Poland

## Abstract

In many countries, including Poland, the main problem with transplantation is the insufficiency of organ donors in relation to the demand for organs. Hence, the common aim globally is to increase the pool of donors. The prolonged survival of patients after transplantation, with respect to the survival time of patients on dialysis, makes the search much more intense. After the recourse of expanded criteria donors (ECD), the next step was obtaining kidneys from donors after irreversible cardiac death (DCD). Therefore, based on Dutch, British, and Spanish experience, it can be hypothesized that the introduction of DCD procedures in countries that have not launched these programs and the improvement of DCD procedures may shorten the waiting time for organ transplantation globally. The legal basis for the procurement of organs after irreversible cardiac arrest came into existence in Poland in 2010. Previously, such organ procurements were not in practice. Since 1984, when Poland published irreversible cardiac arrest as a criterion of brain death, it became the only way to determine death prior to the procurement of organs. The aim of this report was to evaluate the results of the first 19 transplantation cases involving harvested kidneys from donors after cardiac arrest, which was irreversible and clinically confirmed, without any doubt as per the ethical protocol of DCD. Understanding, support, and public perception are essential for this program's initiation and maintenance.

## 1. Introduction

A recent global problem is the persistent shortage of the kidney pool for transplantation. Despite increases in kidney transplantation from expanded criteria donors (ECD) and living related donors (LRD) in the last decades, the supply of donor kidneys remains insufficient [1]. Shortage of organs for transplantation connected with the promising results of organs transplanted from donors after cardiac death has evolved the use of donors after irreversible cardiac death (DCD) into routine practice in many western countries and accounts for 16.5% of organ transplantations in the US (based on OPTN Data of March 2016) and up to 52% in Netherlands [2–5]. In some Asian countries and in Japan, DCD constitutes the main source of organs [6]. The First International Workshop on DCD held in Maastricht in 1995 described four categories of DCD, depending on the irreversible cessation of circulatory and respiratory functions [5]. Based on the Maastricht categories, uncontrolled DCDs are Types I (dead on arrival) and II (unsuccessful resuscitation), while controlled DCDs are Types III (awaiting cardiac arrest) and IV (cardiac arrest occurring with brain death). The European countries that actively perform DCD differ in their protocols. The highest rate of controlled DCD is recorded in the United Kingdom, Belgium, and the Netherlands while uncontrolled DCD mainly is described in France, Latvia, and Spain [7]. In recent years, in the Council of Europe, 10 out of 27 participating countries confirmed DCD activity. For a long time, Poland and 9 other countries such as Cyprus, Estonia, Luxembourg, Norway, Portugal, Romania, Slovak Republic, Slovenia, and Sweden have been planning to start the DCD program [8, 9]. Successful European uncontrolled donation after circulatory determination of death (UDCDD) programs rely on legislation permitting organ procurement without consent. The legal basis for the procurement of organs from donors after irreversible cardiac arrest has existed in Poland since 2010, but it was in May 2015 when the first cases occurred in the Department of General and Transplant Surgery, Clinical Hospital of Poznan University of Medical Sciences [10, 11].

The procurement was initiated according to a standardized protocol, designated to select and manage kidney DCD. Based on this, the implemented DCD program describes as eligible for organ retrieval, uncontrolled Maastricht Types I and II–the UDCDD protocol. In Poland, Maastricht III donors must be excluded based on the law.

## 2. Materials and Methods

Between May 2015 and April 2017, in the Clinical Hospital of Poznan University of Medical Sciences, 10 non-heart-beating donors (DCD) Maastricht Types I/II were accepted for organ retrieval.

The acceptable criteria for DCD were as follows: (i) known identity; (ii) age less than 60 years; (iii) time from cardiac arrest to cardiopulmonary resuscitation (CPR) less than 30 min; (iv) donors' warm ischemia time less than 180 min; (v) prosecutor's consent; (vi) negative history of diabetes mellitus, uncontrolled hypertension, malignancy, renal disease, extensive trauma, or systemic sepsis.

After retrieval the kidneys were put into the WAVES system which provides controlled pulsatile kidney perfusion using oxygenated hypothermic physiologic solutions and monitors, displays, trends, and saves important perfusion parameters, including perfusate flow, temperature, pressure, and renal resistance. The temperature and resistance to flow were constantly recorded. One of 20 kidneys was not accepted for transplantation because of significantly high renal resistance (RI>0,4).

During the 2-year period from May 10, 2015, to April 21, 2017, a total of 19 renal transplantations of DCD kidneys involving 11 male and 8 female recipients were performed. The ages of the recipients ranged from 32 to 69 years (median, 50.9 years). All the patients were dialyzed before the transplantation for 9 to 120 months (median, 30 months). The causes of kidney failure are shown in Table 1. For 4 patients, it was the second kidney transplantation. In the previous transplantation procedure kidneys derived from DBD donors.

Patients were informed that the kidneys were from deceased donors after irreversible cardiac arrest and they signed a specially prepared consent form. HLA typing and cross-matching were routinely performed before the DCD kidney transplantation.

### 2.1. Ethics Committee Approval

Renal transplantation of DCD kidneys is a standard procedure and does not need the ethics committee approval.

### 2.2. Immunosuppressive Protocols

Recipients were immunosuppressed using a triple-therapy regimen at the beginning of the DCD transplantation program. Patients were given Simulect (Basiliximab 20 mg) intravenously as induction therapy prior to the transplantation and on the 4th day after kidney transplantation. Methylprednisolone (intravenous) 500 mg, 250 mg, and 125 mg were administered for 3 days after the transplantation, consecutively. Prednisone was introduced on the 3rd day after transplantation (20 mg/day). Tacrolimus (for 11 patients) was started at a dose of 0.1 mg/kg/day with mycophenolate mofetil (1.0-1.5 g/day) on day 0 and was adjusted to maintain whole blood levels in the range of 8 to 14 ng/mL, in the first 3 months after transplantation, and 5 to 12 ng/mL, thereafter. Cyclosporine (for 5 patients) was started at a dose of 10 mg/kg/day with mycophenolate mofetil (2.0-3.0 g/day) and the dose was adjusted to maintain whole blood levels in the range of 250 to 350 ng/mL. Subsequently, this regimen, according to the practice of more experienced centers, was based on two drugs without mycophenolate mofetil. Everolimus was started with tacrolimus and steroids in the remaining 3 cases. These protocols differed due to comorbidities, PRA level, and coexisting precancerous state.

### 2.3. Follow-Up

The follow-up period was 10 to 28 months. The results were obtained directly from the transplant outpatient clinic visited by the patients and via a telephone survey.

## 3. Results

There were 6 male and 4 female cadavers among the donors. The age of the donors ranged from 28 to 62 years (median 50 years).

CPR lasted for 35 to 125 min, with a 5-min “no touch time” period. External cardiac massage was performed using mechanical chest compression (Lucas II) in 9 cases and manually in the first case. In 3 of the 10 procurements, normothermic extracorporeal membrane oxygenation (nECMO) was used. The average total warm ischemic time (WIT) was 167.1 min. Seventeen of 19 transplanted DCD kidneys were preserved using cold continuous pulsatile preservation perfusion with an IGL-1 liquid. (Table 2)

### 3.1. Early Graft Function and Rejection Rates

Two deaths were recorded: 1 female patient, due to severe pneumonia in the 54^th^ postoperative day, and a male patient, due to a large retroperitoneal hematoma in the 23^th^ postoperative day. The deaths were not related to the type of donation.

Three NHBD kidney transplants did not function satisfactorily, giving an overall primary nonfunction rate of 15.8%. In the 2 patients who died, it was not known whether the allografts were viable at the time of death. The acute rejection rate was 5.3%. This single case was successfully treated with solu-medrol pulses.

Delayed graft function was observed in all the recipients. The number of necessary hemodialysis after transplantation ranged between 2 and 18 (average, 8.4). The number of transfused units of RBC concentrate ranged between 2 and 18 (median, 6).

The urological complication rate was 5.3%. Urological complication was observed in a male patient who was operated on several times because of a large retroperitoneal hematoma. After one of the surgical procedures, urine leak from the ureterovesical anastomosis appeared.

### 3.2. Graft Survival

One-year graft survival rate was 68.4%. Excluding the early deaths, the rate is 78.9%. Four graftectomies were performed: 3 because of unsatisfactory graft function and 1 because of surgical postoperative complications leading to graft infection. 3 patients required chronic dialysis. No rejection was found in performed graft biopsies. In addition, urinary tract infections recurred.

### 3.3. Late Graft Function

**Figure 1:**
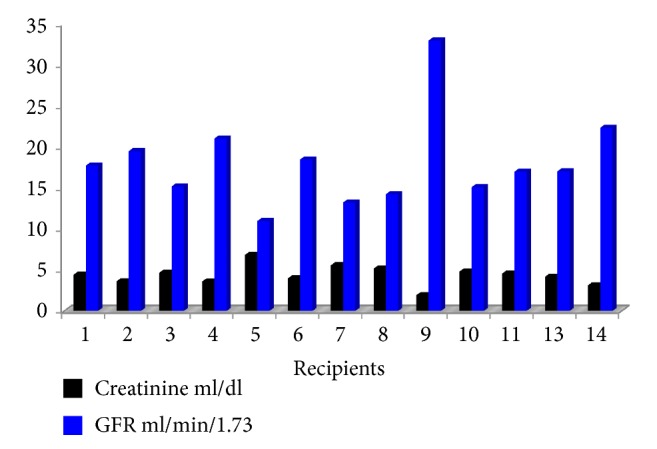
Creatinine and GRF level at the end of posttransplant hospitalization.

The baseline serum creatinine levels (at the end of posttransplant hospitalization) are shown in Figure 1. They ranged between 1.93 and 5.57 mg/dL (median, 4.12 mg/dL). GFR levels ranged between 11 and 36 mL/min/1.73 m^2^ (median, 17)

**Figure 2:**
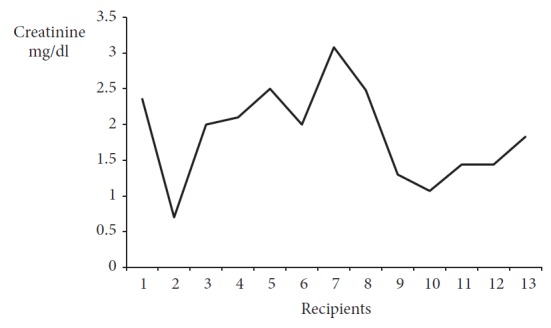
Minimal creatinine level.

All the DCD kidneys improved functionally in 1 to 3 months after the recipients' discharge from the hospital and remained stable in the follow-up period. The minimal creatinine levels ranged between 0.7 and 3.04 mg/dL (median 2) (Figure 2).

GFR levels ranged between 24 and 98 mL/min/1.73 m^2^ (median, 38).

**Figure 3:**
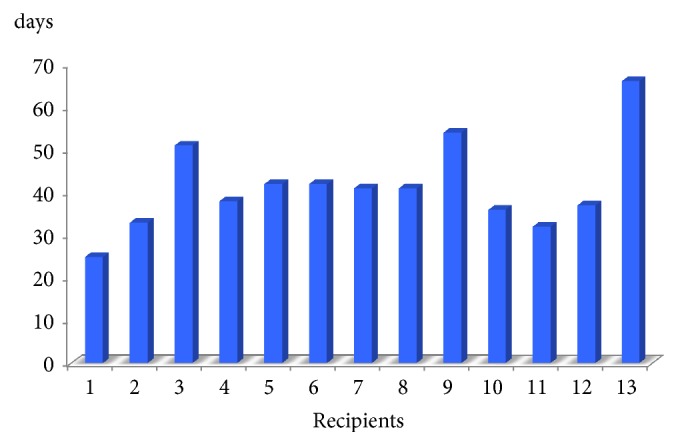
Hospitalization.

None of the patients required reoperation. All of them are very satisfied with the transplanted kidneys, although they admitted that the prolonged hospitalization (Figure 3) and waiting for the transplanted kidney to exhibit good renal function were difficult.

### 3.4. Contribution of DCD Kidneys

**Figure 4:**
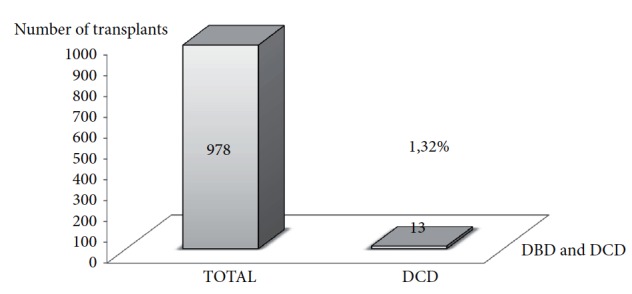
The contribution made by DCD kidneys to the transplant rate in Poland in 2016.

Figure 4 shows the contribution made by DCD kidneys to the transplant rate in Poland in 2016. In 1 year, 13 DCD kidney transplants were performed, leading to 1.32% of a total of 978 kidney transplants.

## 4. Discussion

One-year graft survival rate was 68.4%. The results were understated due to 2 deaths not related to the type of donation. All the DCD kidneys improved in functionality in the 1 to 3 months following the recipients' discharge from the hospital and remained stable during the follow-up period. Moreover, none of the patients needed to be reoperated on; instead, they were satisfied with the outcome of the procedure. Although the results are not good enough comparing with other experienced centers, they show honestly initial difficulties in the development of a non-heart-beating donor program. The troubles with kidneys derived from UDCD were common in transplantation teams starting this program [12, 13].

For dialysis patients with end-stage renal failure, transplantation may provide additional years of life and an improvement of quality of life. The decrease in the number of organ procurements from donors after brain deaths observed in the previous year in Poland is associated with a reduction in mortality from traffic accidents and cerebrovascular diseases. In some other countries, it has remained at a similar level as in the UK, for example, or has decreased as in Spain, for example [13, 14].

The median waiting time before transplant for adult patients was about 9 months. Meeting the ever-growing difference between the demand for organs and their harvesting involves the use of ECD, considered less than optimal, involving older donors, donors with comorbidities who show deteriorated long-term graft survival, and donation after cardiac death [15].

Early graft loss is more frequent among DCD and ECD kidney recipients than among DBD kidney, and DCD kidneys are more susceptible to cold ischemic injury and have a higher incidence of delayed graft function than DBD ones. Short and medium transplant outcomes were similar in the DBD and DCD groups [16–19]. It is also emphasized that kidney transplant patients following uncontrolled DCD recover renal function at a slower rate than recipients of controlled DCD [20]. Moreover, several papers indicate similar long-term outcomes between expanded-criteria DCD (ECDCD) and DBD (ECDBD) donors. Early graft loss in DCD recipients is associated with a higher incidence of primary nonfunction and acute vascular occlusion because DCD kidneys are more vulnerable to ischemia-reperfusion injury [21]. A higher percentage of early graft loss due to acute thrombosis may be linked to poor quality vessels and endothelial activation in DCD donation [22]. It is therefore important to seek a procedure that improves the quality of procured organs and shortens the time of warm ischemia. Successful UDCDD programs exhibited optimal results when warm ischemic time was up to 120 min [23], but the success was achieved at time up to 180 min [24]; thus, experts suggest considering only witnessed cardiac arrests or patients with recent cardiac activity (any rhythm other than asystole) during resuscitation. To prevent microvascular clotting, initiation of preservation with heparin infusion, chest compression, and assisted ventilation should occur soon after death determination. In addition, nECMO is selected as the preservation method due to its protective and reparative properties [25–28]. Based on many studies, the currently implemented procedure in our hospital involves the use of nECMO perfusion of organs, which can cause minor injury owing to ischemia-reperfusion injury and improve the quality of procured organs [28, 29]. Additionally, the cold continuous pulsatile preservation perfusion will use WAVES and an IGL-1 liquid. The results should be better by ensuring lower renal resistance RI<0,4.

The UDCDD program has qualified donors who meet the following criteria: age 18-60, known personal data, excluded potentially reversible causes of cardiac arrest, known CPR time <30 min or documented electrical activity of the heart leading to a cardiac arrest (suggesting a recent cardiac arrest), and without exclusive factors: CPR performed over 30 minutes after the arrest, massive hemorrhage, renal failure (e.g. dialysis access), liver disease (e.g. jaundice, ascites), drug addiction, homelessness, cancer and infections, severe trauma, amputations in the course of vascular disease, or period of severe hemodynamic insufficiency longer than 1 hour (e.g. peripheral edema) before death.

The DCD procedure has raised ethical controversies not only in Poland, which is important in the protocol of organ recovery and the procedure followed by the transplantation team. The standard way to avoid conflicts of interest, loyalty, and obligation is that members of the transplant team, acting on behalf of potential recipients, are not involved in CPR or diagnosis of patient death. This so-called “hands-off” period with separation of roles for teams is indispensable for public trust and the development of the organ donation program [30, 31].

Every year in Poland, approximately 600 kidney procurements from donors after brain death are performed. The number of transplants from living donors is small, approximately 55 per year [30]. The program of organ donation after cardiac arrest is a strategy to increase the pool of donors while shortening the waiting time for transplant patients on waiting lists. However, this group of donors is not fully utilized worldwide, especially in Poland. The protocol established in the Department of General and Transplant Surgery of Poznan University of Medical Sciences might be helpful for other transplant teams in Poland to start a program.

## 5. Conclusion

Our data show convincing results concerning the first 19 cases of UDCDD in Poland. The DCD program involving uncontrolled donors is challenging for transplant coordinators, hospital transplant teams, and out-of-hospital emergency services. Continuous effort by many people, with nationwide information campaigns for the acceptance of the donation of organs after cardiac death by the Polish population, is crucial for future success in increasing the pool of organs donors. It seems that only the establishment of a program of donor renal transplantation from DCD Maastricht Types I and II is a promising option to reduce the number of potential recipients on waiting lists in Poland. These campaigns may also influence Polish law and change the legislation to allow for DCD Maastricht Type III.

## Figures and Tables

**Table 1:** Causes of renal failure.

Cause of renal failure	Number of recipients

Glomerulonephritis	3
Diabetic nephropathy	3
Hypertensive nephropathy	2
Polycystic kidney disease	2
Focal segmental glomerulosclerosis	2
Interstitial nephritis	2
Vasculitis	1
Others	4

**Table 2:** Donors' characteristics.

Sex	Age (in years)	Cause of death	CPR on arrival (min)	CPR in the hospital (min)	No touch time (min)	Chest compression	WIT

M	45	Myocardial infarction	35	57	5	Manual	169
K	28	Asphyxia	20	76	5	Mechanical Lucas II	164
M	62	Myocardial infarction	30	75	5	Mechanical Lucas II	175
M	53	Myocardial infarction	5	86	5	Mechanical Lucas II	145
M	49	Myocardial infarction	66	30	5	Mechanical Lucas II	160
K	58	Myocardial infarction	84	41	5	Mechanical Lucas II	203
K	45	Myocardial infarction	46	47	5	Mechanical Lucas II	158
K	41	Myocardial infarction	53	46	5	Mechanical Lucas II	177
K	54	Myocardial infarction	35	37	5	Mechanical Lucas II	160
M	47	Myocardial infarction	12	23	5	Mechanical Lucas II	160

## Data Availability

Data supporting the conclusion of the study are available in patient's medical records and Polish transplantation records.
